# A Freely Accessible, Anonymous Online Treatment for Social Anxiety: Protocol for a Partially Randomized Patient Preference Trial

**DOI:** 10.2196/77573

**Published:** 2025-11-05

**Authors:** Stefanie Arnold, Michael Felix Vogt, Johanna Boettcher, Friederike Fenski, Dajana Šipka, Malte Elson, Thomas Berger

**Affiliations:** 1 Department of Clinical Psychology and Psychotherapy Institute of Psychology University of Bern Bern Switzerland; 2 Department of Psychology of Digitalization Institute of Psychology University of Bern Bern Switzerland; 3 Department of Clinical Psychology and Psychotherapy Psychologische Hochschule Berlin Berlin Germany

**Keywords:** internet-based cognitive behavioral therapy, social anxiety, anonymity, open access, digital mental health intervention, digital health, eHealth

## Abstract

**Background:**

Many people with mental health problems do not receive the care they need. Digital mental health interventions have been shown to be effective in many trials and offer a promising way to reach more people in need. However, their uptake and use remain limited, in part due to concerns about data privacy. However, these concerns may not be equally significant for all users.

**Objective:**

This trial aims to evaluate the efficacy of an anonymous online self-help tool for social anxiety that processes no personal data and has some disadvantages in terms of usability compared to an otherwise identical, nonanonymous evidence-based tool. Furthermore, the trial will investigate user preferences regarding the 2 program versions and evaluate the impact of these preferences on treatment outcomes.

**Methods:**

In this partially randomized patient preference trial, 2 versions (anonymous vs nonanonymous) of the same previously researched program will be compared among 452 participants with increased levels of social anxiety. Half of the participants will be randomized to one of the program versions, whereas the other half will be assigned to their preferred version. The primary outcome is social anxiety symptoms. Secondary outcomes include depression, stigma of mental illness, and quality of life.

**Results:**

Recruitment started in September 2024. As of the date of manuscript submission in May 2025, over 350 participants have been included in the study. Recruitment ended in September 2025, and results are expected to be available and published in 2026.

**Conclusions:**

The results of this trial will determine whether anonymously accessible interventions, which could easily be made freely available, are less efficacious than their nonanonymous counterparts and will explore the impact of user preferences on treatment outcomes. The findings could contribute to making digital interventions more accessible and tailoring interventions to individual preferences.

**Trial Registration:**

ClinicalTrials.gov NCT06465589; https://clinicaltrials.gov/study/NCT06465589

**International Registered Report Identifier (IRRID):**

DERR1-10.2196/77573

## Introduction

As our lives become increasingly digitalized, one of the biggest challenges of our time is successfully transforming our health care system accordingly. The need for digitalization is especially great in the treatment of psychological issues, where access to care remains a significant issue that could be mitigated with digital solutions. A considerable proportion of individuals with psychological issues do not receive the necessary treatment, with reasons including shortages in personnel and limited awareness of treatment options [1]. Therefore, digitalization is crucial as it offers opportunities to reach more people than traditional methods such as face-to-face psychotherapy.

Digital mental health interventions (DMHIs) have been developed to address a wide range of psychological issues. These interventions can be either guided, involving therapist contact, or unguided, where individuals engage with the content independently. Although guided interventions have been shown to be more effective than unguided interventions [2], unguided interventions have also demonstrated effectiveness as low-threshold options for individuals who might not seek treatment otherwise [3]. Although many of these DMHIs are being evaluated in research and implemented in routine practice [4], the acceptance and use of such interventions remain relatively low among health care personnel and potential users [5-7]. Surveys with physicians, psychotherapists, and patients show that some of the greatest barriers to using online interventions are privacy and data security concerns [8,9]. Notably, the study by Batterham et al [8] is representative of the adult population in Australia, thereby highlighting that these concerns are prevalent among the public, including individuals who may not proactively seek DMHIs. In addition, the possibility to stay anonymous is reported as one of the chief factors influencing participation in DMHIs [8]. This underscores the importance of addressing privacy and data security in the development of such interventions, particularly in efforts to maximize accessibility and reach a broad audience.

These data privacy concerns are not unfounded as digital mental health applications have been found to be susceptible to data breaches and unauthorized access [10]. Beyond security vulnerabilities, there is also a risk that personal health data may be used for commercial purposes. For example, reports suggest that the online therapy provider BetterHelp shared user data with technology companies for targeted advertising [11]. Such practices not only compromise user trust but also raise ethical concerns about the commodification of sensitive health information, potentially deterring individuals from seeking digital mental health support [12].

Many DMHIs collect personal data such as names, email addresses, and symptom severity [8]. This is typically done to create a personalized user space through log-in access, allowing user inputs to be stored and later used—either by the users themselves to track progress or by the program to adapt the treatment based on individual information. However, evidence-based interventions can convey psychological knowledge, strategies for recovery, and practical exercises without requiring user data collection. While some mental health information is available on freely accessible websites where no user registration is required [13], the literature suggests that the quality and helpfulness of online mental health information vary considerably [14]. In addition, many of these websites typically provide general psychoeducational content rather than structured, interactive interventions. While such general resources can serve as initial support, comprehensive DMHIs with interactive elements—such as progress tracking, reminders, or tailored exercises—are often considered more effective [15].

Anonymity in DMHIs presents both advantages and limitations. Anonymous DMHIs, meaning programs that do not store user data, could lower barriers to access by alleviating privacy concerns, thereby increasing use among individuals hesitant to disclose personal information. In addition, such programs eliminate the need for secure data storage, reducing technical and regulatory burdens. However, anonymity restricts certain intervention features such as personalized feedback, progress tracking, and therapist interaction. From a research perspective, evaluating intervention efficacy becomes more challenging without user identifiers, limiting the ability to track long-term outcomes. Thus, the development of anonymous DMHIs requires balancing privacy protection with an effective intervention design. It is important to note that, while such anonymous DMHIs do not store user data in a personally identifiable manner, they may still use nonidentifiable technical data (eg, essential cookies) required for functionality. In contrast to conventional, nonanonymous programs, anonymous DMHIs are characterized by their inability to attribute data to specific individuals.

In summary, while anonymity in DMHIs may increase accessibility, it also introduces trade-offs that could impact intervention efficacy. This raises some key questions: are anonymous DMHIs less efficacious compared to conventional, nonanonymous programs? If presented with the choice between an anonymous and nonanonymous version of the same program, would users, given the privacy and data security concerns reported in previous research, prefer the anonymous version? This study aims to address these questions within the context of social anxiety (SA). While SA refers to heightened levels of anxiety in social situations and exists on a continuum within the general population, SA disorder (SAD) is a clinical condition characterized by an intense and persistent fear of social evaluation, often leading to significant distress and avoidance behaviors [16-18]. SAD is a condition for which DMHIs have been extensively researched [19-21]. SA and SAD are particularly relevant for DMHIs as individuals with SA and SAD often experience heightened fear of social evaluation, which can make seeking traditional health care services daunting [22]. By examining an anonymous version of the self-help intervention JOURNeY that is based on an evidence-based self-help intervention [23-27], this study will investigate the efficacy of an anonymous DMHI compared to a standard nonanonymous version. Furthermore, user preferences regarding anonymity and their potential impact on treatment outcomes will be explored. Understanding these aspects could inform future developments in digital mental health, particularly in optimizing accessibility while maintaining intervention effectiveness.

## Methods

### Study Design

**Figure 1:**
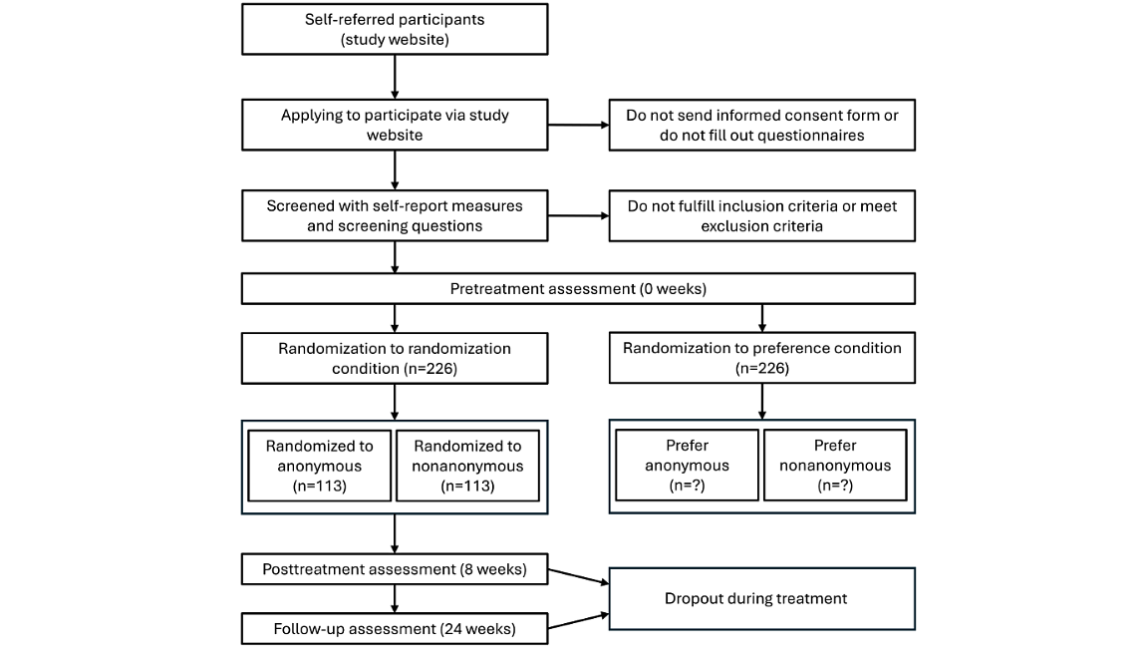
Design of the study and expected participant flow.

This study is part of the larger research project PRIVATE (Preference Research: Investigating Variations of Anonymity, Transparency, and Efficacy in Digital Health Applications) and is a single-center, partially randomized patient preference trial. There are 2 program versions: anonymous (a tool that does not allow user identification) versus nonanonymous (a tool that allows user identification). Participants are assigned to one of these versions either based on their preference or through randomization. Data are collected at 3 main time points: before the intervention, after the intervention, and at follow-up. This results in a 2 × 2 × 3 design with anonymity (anonymous vs nonanonymous) and allocation method (randomized vs preference) as between-subject factors and time (preintervention time point, postintervention time point, and follow-up) as a within-subject factor. Figure 1 shows the study design and participant flow.

### Ethical Considerations

The PRIVATE study has been approved by the Ethics Committee of the Canton of Bern, Switzerland (2024-00842). Interested people are provided with written study information, where potential risks and benefits are explained. No compensation is offered, and potential participants are informed that they can withdraw from the study at any time without negative consequences. Potential participants provide written consent, where they sign 1 or 2 consent forms. The first and mandatory one is the consent form to participate in the study, and the second optional form concerns consent to use of their encrypted data for future research. Once written consent is obtained, participants can be enrolled in the study. Participant data are collected and handled electronically in REDCap (Research Electronic Data Capture; Vanderbilt University) [28,29], where they can only be accessed by authorized personnel of the study team. Data are stored in encrypted form and are identifiable only by a code that is not linked to the participant’s identity. Data are saved on a protected server of the University of Bern (the firewall-protected Network Attached Storage Unit server of the Institute of Psychology). At no time are the participants’ names and unambiguous personal codes saved together. The code allocation form is saved in a safely encrypted file on an encrypted and protected server of the University of Bern. After the completion of the study, data will be completely anonymized.

### Participants

We will recruit a total of 452 adults with increased SA symptoms, as indicated in the Power Analysis section. Therefore, in the randomization arm, there will be 113 participants per program version. The participant numbers in the preference arm will depend on the participants’ indicated preferences. Candidates will be included if they (1) are aged ≥18 years; (2) return the informed consent form; (3) have access to the internet; (4) have a smartphone, PC, or tablet; and (5) exceed cutoff scores in at least one of the 2 SA measures (>22 points on the Social Phobia Scale [SPS] or >33 points on the Social Interaction Anxiety Scale [SIAS] [30,31]). A formal diagnosis of SAD is not required, and no clinical interviews will be conducted with potential participants. Conducting in-depth and personal interviews would be incongruent with the nature of anonymous DMHIs as such procedures are rarely part of routine practice, where users typically access these tools independently and without previous interaction.

Candidates will be excluded from the study if they (1) report acute suicidality at the beginning of the study (≥2 points on suicide item 9 of the Patient Health Questionnaire–9 [PHQ-9] [32,33]) or (2) report having previously been diagnosed with psychotic symptoms or bipolar disorder.

### Recruitment

Participants will be recruited from the community through internet forums, newspaper articles, social media (eg, Facebook), and our study website in German-speaking countries. To accelerate the recruitment process, the link to the trial will also be publicized using online advertisements, such as Facebook ads, Instagram ads, and Google ads.

### Treatment

#### Overview

The interactive self-help program JOURNeY is grounded in the established cognitive behavioral framework for treating SA as proposed by Clark and Wells [34] and Stangier et al [17]. Its efficacy has been supported by multiple studies [19,23-27,35]. JOURNeY comprises 4 primary therapeutic components: psychoeducation, cognitive restructuring, attention training, and exposure. The content of the 4 main modules and 2 supplementary modules will be explained further in the following subsections.

Participants are advised to use the program for approximately 50 to 60 minutes per week and work on 1 module per week for the first 6 weeks and repeat modules during the last 2 weeks of the intervention period. However, this is only a recommendation, and participants can use the program as they like.

#### Psychoeducation

This module provides comprehensive, evidence-based information on the mechanisms sustaining SA, such as the interplay of negative thoughts, self-focused attention, anxiety, and safety and avoidance behaviors. It encourages participants to build a personalized understanding of how these factors contribute to their own experiences and maintenance of SA. To deepen the participants’ knowledge of SA, they are asked to answer questions about the module content. In addition, participants are instructed to systematically identify and document specific social situations and stimuli that they perceive as threatening or anxiety provoking.

#### Cognitive Restructuring

Participants are guided in recognizing and altering maladaptive beliefs through structured exercises, including keeping a thought diary to capture negative thought patterns and actively practicing techniques to generate more balanced and adaptive interpretations.

#### Attention Training

This component focuses on shifting self-focused attention. Through various video- and audio-guided tasks, participants practice strategies to redirect their focus outward. These include telling a story to a video of a person and focusing on them instead of themselves. This reduces the intense self-monitoring typical in SA and, thereby, interrupts some of the cognitive and emotional processes that maintain the disorder [36].

#### Exposure

Participants are encouraged to systematically engage in real-life exposure tasks. This module involves planning, executing, and reflecting on these exercises using an exposure diary, along with reducing safety behaviors (eg, avoiding eye contact or mentally rehearsing conversations), which typically maintain anxiety. Afterward, participants are asked to reflect on their experiences and what they learned from them.

#### Additional Modules

In addition, there is an introductory module discussing motivation and a conclusion module for relapse prophylaxis, which encourages participants to keep practicing and using techniques they learned in the program in their daily lives. The program will be delivered in an unguided format, which has been shown to be effective in various previous studies [19,24,35], to ensure anonymity and comparability across the 2 program versions, which are introduced in the following section.

#### Program Versions

Both versions of the program are accessible through a standard web browser and provide the same core content. The key difference lies in the presence or absence of account creation and how personal entries made by participants are handled. In the anonymous version (JOURNeY_A), no account or registration is required. To protect anonymity, the sections of the program that involve personal input are provided as a downloadable PDF. Participants can complete and save these entries locally on their own devices. In the nonanonymous version (JOURNeY_NonA), participants create an account, allowing them to enter personal reflections directly into the program and revisit them later. While both versions of the self-help program use cookies for essential functionalities, such as session management and ensuring smooth operation of the platform, these cookies do not track users’ activities or collect personal data, meaning that they do not facilitate the tracking or identification of users in any way.

To account for the obstacle of collection of personal information that is met when assessing such an anonymous DMHI, JOURNeY_A itself will be usable without an account, but within the study, the participants will still regularly receive symptomatology questionnaires to fill out outside of the program facilitated through a data collection platform. This compromise allows us to monitor and assess outcomes to be able to draw conclusions about the efficacy of the program while still maintaining anonymity for users in the program itself.

### Procedures

After receiving the study information and returning the signed informed consent form, the candidates will be screened for eligibility using the self-report measures described in the Instruments section. Participants will additionally be shown brief descriptions of both program versions during screening and asked to select their preferred option. If eligible, the participants will be assigned to a condition. The randomization will be performed in 2 steps to ensure correct condition allocation. In a first step, eligible candidates will be randomized with equal probability to either the randomization or preference condition. In a second step, the participants in the randomization condition will be randomized again with equal probability to one of the program versions (anonymous vs nonanonymous) regardless of previously expressed preference. The participants in the preference condition will be assigned to their preferred program version. Thus, the main effect of the treatment can be estimated, but it is also possible to explore preferences and their possible effects through the preference arm. The first randomization will be conducted using the built-in randomization tool in REDCap [28,29], where all the data will be collected, whereas the second randomization will be conducted by an external researcher with a randomization list created using randomly permutated blocks. An external researcher has to be involved in the randomization as REDCap can only perform one randomization per participant. The condition allocation will be unknown to investigators.

The intervention period, during which participants are asked to use the self-help program, will be 8 weeks. The duration of the study for 1 participant will be 24 weeks. There are 3 main measurement time points during this period: at the beginning (preassessment), after 8 weeks (postassessment), and after 24 weeks (follow-up assessment). In addition, there are 2 minor measurement points, after 3 and 6 weeks, where only preference and other measures unrelated to this part of the study will be assessed. The questionnaires will be sent out automatically through REDCap [28,29] via email at the corresponding time points.

Suicidality is monitored using item 9 of the PHQ-9 at baseline (week 0), the postintervention time point (week 8), and follow-up (week 24). If elevated suicidality is reported at any time point, the study team will reach out to the participant to provide appropriate crisis resources and encourage help seeking. Participants are informed that they may contact the study team at any time if they experience distress or wish to withdraw.

### Instruments

#### Overview

A summary of the instruments and time points of assessment can be found in Table 1.

**Table 1:** Variables, instruments, and time points of assessment.

Dimension			Instrument		Authors (German version)		Time points
**Primary outcome measure**							
	Social anxiety symptoms	SPS^a^ and SIAS^b^		Stangier et al [31]		Before the intervention, after the intervention^c^, and FU^d^	
**Secondary outcome measures**							
	Depressive symptoms	PHQ-9^e^		Gräfe et al [33]		Before the intervention, after the intervention, and FU	
	Quality of life	SF-12^f^		Gandek et al [37]		Before the intervention, after the intervention, and FU	
	Internalized stigma	ISMI^g^		Sibitz et al [38]		Before the intervention, after the intervention, and FU	
	Attitudes toward help seeking	IASMHS^h^		Kessler et al [39]		Before the intervention, after the intervention, and FU	
	Personality functioning	LPFS-BF^i^		Spitzer et al [40]		Before the intervention, after the intervention, and FU	
	Preference	Custom assessment		—^j^		Before the intervention, mid1^k^, mid2^l^, after the intervention, and FU	

^a^SPS: Social Phobia Scale.

^b^SIAS: Social Interaction Anxiety Scale.

^c^8 weeks after baseline.

^d^FU: follow-up (24 weeks after baseline).

^e^PHQ-9: Patient Health Questionnaire–9.

^f^SF-12: 12-Item Short Form Health Survey.

^g^ISMI: Internalized Stigma of Mental Illness scale.

^h^IASMHS: Inventory of Attitudes Toward Seeking Mental Health Services.

^i^LPFS-BF: Level of Personality Functioning Scale–Brief Form.

^j^Not applicable.

^k^3 weeks after baseline.

^l^6 weeks after baseline.

#### Primary Outcome

The primary outcome measure is symptoms of SA at the posttreatment time point (ie, after 8 weeks). It will be measured using the companion scales SPS and SIAS [30,31]. Both self-report measures are frequently used in studies on SAD, and they assess fears of being judged by others during performance-related (SPS) and interaction-related (SIAS) social situations. They have been described as valid, reliable, and useful for clinical as well as research purposes. Good values of internal consistency have been found for the German versions of the scales (Cronbach α=0.94 for both scales [31]). Both scales consist of 40 items that are rated on a 5-point Likert scale (0=“not at all”; 4=“extremely”), with higher scores indicating higher levels of SA. For the analysis, the composite score will be used by simply averaging the *z* scores of the SIAS and the SPS sum score, which has been recommended for continuous variables and done in similar previous research [27,41,42].

#### Secondary Outcomes

##### Overview

Beyond primary treatment outcomes, this study investigates how measures of mental well-being, such as depression severity and quality of life, change during the study. It additionally assesses whether other outcomes, such as internalized stigma, attitudes toward help seeking, and personality functioning, may play a role in shaping preferences for anonymity. These secondary outcomes could help identify for whom and under which conditions anonymous digital interventions may be particularly beneficial.

##### PHQ-9 Instrument

This widely used self-report measure assesses symptoms of depression with 9 items that correspond to the symptoms of major depression described in the *Diagnostic and Statistical Manual of Mental Disorders, Fifth Edition* [18], on a 4-point Likert scale [32,33]. Higher scores indicate more severe depressive symptoms. The PHQ-9 has shown good internal consistency as well as good sensitivity and specificity [33,43,44].

##### 12-Item Short Form Health Survey

This self-report instrument measures quality of life. It has 2 subscales measuring physical and mental aspects of health-related quality of life with a total of 12 items. The 12-Item Short Form Health Survey has shown good psychometric properties, roughly equivalent to those of the 36-Item Short Form Health Survey, and is widely used as an estimate of general quality of life [37,45].

##### Internalized Stigma of Mental Illness Scale

This 29-item self-report scale [38,46] measures the internalized stigma of mental illness. It is widely used in various contexts and consists of 5 subscales: alienation, stereotype endorsement, discrimination experience, social withdrawal, and stigma resistance. It has shown good internal consistency and good test-retest reliability [38]. Often, the stigma resistance subscale is excluded in analyses as some researchers have suggested that another construct be measured using this subscale [47]. As suggested by these researchers, preliminary factor analysis will be conducted to decide whether to include this subscale in the total score.

##### Inventory of Attitudes Toward Seeking Mental Health Services

This 24-item self-report scale [39,48] measures the participants’ attitudes toward seeking help for mental health issues. It is an adaptation of an older scale [49] and has 3 subscales. This scale is used in nonclinical as well as clinical samples. The German version has shown acceptable psychometric properties [39].

##### Level of Personality Functioning Scale–Brief Form 2.0

This 12-item self-report tool [40,50,51] measures the impairment in self-functioning regarding sense of self and interpersonal functioning based on the Alternative DSM-5 Model of Personality Disorders [18]. The questions are answered on a 4-point Likert scale (1=“does not apply at all”; 4=“applies exactly”). It consists of 2 subscales and is a fairly new tool. A thorough validation for clinical samples is still pending, but good internal consistency for the German scale has already been determined [40].

##### Preference

Preference will be assessed by presenting all participants with 2 approximately 150-word texts describing the 2 versions of the program. They will then be asked to indicate their preference and their preference strength by answering the question “How important is this choice to you?” on a 4-point Likert scale (1=“not important at all”; 4=“very important”), as done previously [52]. Participants are only forced to make a choice at preassessment to enable possible condition allocation for all participants that are randomized to the preference condition. At the remaining measurement points, participants can indicate that they have no preference.

##### Engagement

When it comes to engagement, “the more use the better” is not always true [53], especially for internet interventions such as this one, where the participants determine the dosage themselves. However, given that one version will be completely anonymous, it will not be possible to use complex measures of engagement for individual participants in this condition at a technological level. To find a compromise and gain insights into program use, the total number of clicks during the intervention period will be recorded for both program versions and then divided by the number of participants per version, providing an average that can be compared between groups. This has been done before for other internet interventions [54].

### Power Analysis

This study is powered regarding the primary outcome (composite score of the SPS and SIAS) and first study aim (whether anonymous digital interventions are less efficacious than conventional, nonanonymous programs). This means the comparison of the 2 versions in terms of reduction in SA symptoms in the randomized condition. As we expect a superiority of the nonanonymous version, a Cohen *d* value of 0.35 was used as the target effect size between groups as we considered smaller effects clinically insignificant. We conducted a power analysis for Cohen *d*=0.35 with an α level of .05 and a power of 0.80 for the time × group interaction. The sample size was determined using the ANOVA power Shiny app [55], which is based on the R programming environment [56], and the *Superpower* package for R (R Foundation for Statistical Computing). This app performs Monte Carlo simulations tailored to factorial experimental designs, allowing for interaction effects and comparisons across both within- and between-subject factors [55]. For the power analysis, we defined the randomized condition as a mixed 2 × 3 design with 2 program versions (between) and 3 time points (within). For means and SDs of the nonanonymous version, values from another trial with the same basic online program were taken [26], and for the anonymous version, the means were calculated using the aforementioned effect size. The SDs used were the same as in the nonanonymous version, and the correlation of within-subject factors was set at *r*=0.5. For SPS values with 2000 simulations, a total of 113 participants per condition are needed, and for SIAS values, 112 participants are needed per condition to reach 0.80 power for the interaction effect. Therefore, for the 2 randomized arms, 113 participants per arm will be recruited. Although we do not know beforehand how many participants will be in each of the 2 preference arms, we doubled the participants from the randomized arm for the power analysis, resulting in a total of 452 participants across all 4 arms.

### Statistical Analysis

We will evaluate the differences in SA symptom change between the anonymous version (JOURNeY_A) and the nonanonymous version (JOURNeY_NonA) using linear mixed models with the posttreatment assessment as the confirmatory primary end point. Time will be used as the within-subject factor, and condition will be used as the between-subject factor. Fixed effects will be time, condition (JOURNeY_A or JOURNeY_NonA), and the intervention term; the random effect will be the intercept of each participant as every participant is expected to have a different prescore. This analysis accounts for the correlation of residual errors within participants and includes a treatment-by-time interaction term to assess differential symptom change over time [57]. Estimates for each time point will be derived from this model. We will conduct the analysis based on the intention-to-treat principle. The intention-to-treat analysis set consists of all participants in the randomized group regardless of any protocol violations. The inferences about overall program efficacy will only be made based on the randomized arm. For the exploratory preference analysis, appropriate methods such as multiple logistic regression, *t* tests, and chi-square tests will be used depending on the type of data. Comparisons will focus, for example, on participants who received their preferred treatment (preference match) versus those who did not (preference not matched). The amount and pattern of missing data will be reported, and results will be interpreted accordingly in a cautious manner. To allow for comparison across trials, we will also report the standardized mean differences (effect sizes).

## Results

This study was funded in 2023, approved by the Cantonal Ethics Committee of Bern, Switzerland in May, 2024 and recruitment and data collection started in September 2024 and are ongoing as of the submission of this manuscript. As of the submission in May 2025, over 350 participants have been included in the study. Recruitment of the study was finished in September 2025 and data collection is projected to be finished in March 2026. No data has been analyzed yet.

## Discussion

### Principal Implications

Internet interventions can present a low-threshold and effective way of providing evidence-based care. However, low acceptance and use rates highlight the need to design interventions that directly address potential users’ concerns, particularly regarding data security and privacy. Freely accessible, evidence-based, and anonymous DMHIs could play a role in bridging the treatment gap for mental health care. This trial will assess whether an anonymous self-help tool for SA can be efficacious while addressing privacy concerns. In addition, the findings will provide insights into user preferences for anonymous versus nonanonymous versions.

### Limitations

One limitation of this trial is that all measures rely solely on self-report questionnaires without incorporating observer-based measures such as assessments by a mental health professional to evaluate clinical outcomes. As the goal is to evaluate the tool in its intended real-world use, no in-depth diagnostic interviews will be conducted to avoid compromising participants’ sense of anonymity. Second, the preference-based study arm may introduce confounding factors as user preferences might be linked to other characteristics that influence outcomes [58]. However, to still be able to make inferences about the overall efficacy, the randomization arm allows for direct comparison between the 2 program versions. Third, even in the anonymous conditions, participants provide intimate information through questionnaires, which may still affect their perception of anonymity. To mitigate this concern, we emphasize at various stages of participant interaction that only the program is anonymous, whereas the study itself does not guarantee complete anonymity. This approach strikes a balance between exploring the efficacy of an anonymous DMHI and monitoring changes in outcomes of interest. Fourth, it is plausible that individuals who place a particularly high value on anonymity may opt not to participate in the study due to the required disclosure of personal information during study procedures. As a result, this potentially relevant subgroup of the target population may be underrepresented in the sample, limiting the generalizability of the findings. Finally, the inability to accurately measure user engagement with the program is a problem. As there are no individual user data, the only indicator of adherence is the total amount of clicks registered on the website, which we will then divide by the total number of participants with access to the site during the trial to at least obtain some insights into user engagement.

### Future Directions

Future research should examine whether similar anonymous DMHIs can be effective for other psychological conditions. In addition, further investigating user characteristics that influence preferences and engagement could provide valuable insights into optimizing intervention design. Our study data may also offer initial indications of which users benefit most from an anonymous format.

### Conclusions

In conclusion, this trial will have important implications for future internet intervention development regarding degree of anonymity and possibilities of ensuring privacy. Freely and anonymously accessible DMHIs offer a low-threshold alternative to traditional approaches such as face-to-face psychotherapy. These interventions hold significant potential to extend evidence-based care to a wider audience, addressing unmet mental health needs and narrowing the mental health treatment gap.

## Abbreviations

DMHI: digital mental health intervention
IASMHS: Inventory of Attitudes Toward Seeking Mental Health Services
ISMI: Internalized Stigma of Mental Illness scale
LPFS-BF: Level of Personality Functioning Scale–Brief Form
PHQ-9: Patient Health Questionnaire–9
PRIVATE: Preference Research: Investigating Variations of Anonymity, Transparency, and Efficacy in Digital Health Applications
REDCap: Research Electronic Data Capture
SA: social anxiety
SAD: social anxiety disorder
SF-12: 12-Item Short Form Health Survey
SIAS: Social Interaction Anxiety Scale
SPS: Social Phobia Scale

## Data Availability

Data sharing is not applicable to this article as no datasets were generated or analyzed. Following study completion, deidentified data and accompanying documentation will be made available in an open access data repository in accordance with the findability, accessibility, interoperability, and reusability data principles and ethics approvals.
